# A multi-dataset associative analysis of single-cell and bulk transcriptomic profiles in preeclampsia

**DOI:** 10.1515/med-2026-1529

**Published:** 2026-10-26

**Authors:** Bei Liu, Xuena Cui, Xiaofei Wang, Min Zhao, Lin Xu

**Affiliations:** The Affiliated Hospital of Qingdao University, Qingdao, Shandong Province, China

**Keywords:** preeclampsia, immune cell subpopulations, immune infiltration, candidate regulators

## Abstract

**Objectives:**

Preeclampsia is a multifactorial disease involving diverse pathological mechanisms and signaling pathways. However, the specific mechanism of PE is not yet completely elucidated.

**Methods:**

The scRNA-seq dataset GSE173193 and the bulk transcriptomic dataset GSE74341 were obtained from the Gene Expression Omnibus and analyzed separately as complementary datasets. The single-cell dataset was used to explore cellular heterogeneity, inferred intercellular communication, pathway activity, candidate regulons, and transcriptional-state trajectories. The bulk dataset was analyzed using CIBERSORT, GSEA, and GSVA to estimate immune-cell fractions and assess their associations with candidate regulators. Cross-dataset findings were interpreted as complementary association-level evidence rather than direct validation.

**Results:**

Within the analyzed datasets, macrophages/monocytes exhibited relatively high inferred communication activity. FOLR2^+^ macrophages and APOBEC3A^+^ monocytes were identified as candidate subpopulations of interest, while EVX1 and KLF9 were identified as computationally predicted candidate regulators associated with immune and metabolic pathways. In the bulk transcriptomic dataset, CIBERSORT using the blood-derived LM22 reference matrix estimated higher fractions of dendritic cells and resting mast cells and a lower fraction of activated CD4^+^ memory T cells in the preeclampsia samples. These estimates may not fully represent specialized placental or decidual immune-cell populations and should not be interpreted as directly measured changes in placental immune-cell abundance. Given the limited number of biological samples and the lack of detailed clinical metadata, these observations should be regarded as exploratory dataset-level associations that may be influenced by inter-individual, gestational, or other clinical heterogeneity.

**Conclusions:**

This study employed complementary analyses of independent single-cell and bulk transcriptomic datasets to identify exploratory immune-cell and transcriptional associations in preeclampsia. The identified cell populations and candidate regulators provide hypotheses for future investigation but require confirmation in independent cohorts and experimental studies.

## Introduction

Preeclampsia (PE) is a complex obstetric complication that poses significant life-threatening risks during pregnancy, increasing the likelihood of maternal and neonatal morbidity and mortality [1], 2]. Affecting approximately 2–8 % of pregnant women globally, PE typically manifests after twenty weeks of gestation [3]. It is diagnosed by the onset of new hypertension, defined as a systolic blood pressure of ≥140 mmHg or a diastolic BP of ≥90 mmHg, accompanied by either proteinuria or other symptoms such as renal insufficiency, thrombocytopenia, hepatic dysfunction, pulmonary edema, or cerebral/visual disturbances. These symptoms can emerge suddenly and without warning [4], 5]. Understanding the pathophysiological mechanisms of PE is crucial for improving our ability to prevent, manage, and treat this disorder, thereby reducing adverse outcomes for both mothers and fetuses. Despite extensive research into the pathogenesis of PE, a consistent pathological mechanism has yet to be identified [6]. Current studies suggest that vascular endothelial dysfunction, oxidative stress, abnormal placental trophoblast differentiation, and an imbalanced maternal-fetal immune response are key factors in the pathogenesis of PE [7], [8], [9], [10], [11].

The management of preeclampsia primarily involves controlling blood pressure, preventing seizures, and ensuring timely delivery. Common antihypertensive medications include labetalol and nifedipine, while magnesium sulfate remains the first-line treatment for preventing and managing eclamptic seizures [12], 13]. Currently, there is no cure for preeclampsia; delivery of the baby and placenta is the only effective treatment. The timing of delivery depends on gestational age and maternal-fetal health status. Extensive research has focused on improving the prediction, diagnosis, and treatment of preeclampsia. For high-risk groups, administering low-dose aspirin between 12 and 16 weeks of gestation and continuing until 36 weeks significantly reduces the incidence of preeclampsia [14], 15]. Additionally, calcium supplementation (1.5–2 g daily) is effective in preventing preeclampsia in women with insufficient calcium intake [16]. Calcium deficiency during pregnancy can lead to complications such as gestational hypertension, reduced bone mineral density, impaired fetal development, and other adverse outcomes. Maintaining a healthy lifestyle – through weight management, balanced nutrition, and moderate exercise – along with regular prenatal check-ups, is crucial for early detection of hypertension, abnormal proteinuria, and placental dysfunction [17]. The FIGO guidelines stress the importance of increasing awareness of the link between preeclampsia and adverse maternal and perinatal outcomes, as well as its long-term health implications for offspring. All pregnant women are advised to undergo early screening for preeclampsia in the first trimester, using a combination of maternal risk factors and biomarkers. The optimal screening approach includes assessing maternal risk factors, mean arterial pressure (MAP), serum placental growth factor (PLGF), and uterine artery pulsatility index (UTPI) measurements [18].

Recent studies have shown that immune cells are pivotal in the pathogenesis of preeclampsia [11]. Macrophages and monocytes, in particular, have attracted attention due to their regulatory roles in immune responses, inflammatory reactions, and placental immune tolerance [19]. These cells may significantly influence the onset and progression of preeclampsia by modulating the local immune microenvironment and engaging in maternal-fetal immune interactions [20], 21]. Macrophages not only execute inflammatory responses but also interact with other cells by secreting cytokines and growth factors, impacting placental function and angiogenesis [22], 23]. In preeclampsia’s abnormal immune response, alterations in the number and function of macrophages and monocytes are closely linked to placental vascular insufficiency and maternal immune tolerance failure [24], 25].

## Methods

### Data acquisition

The Gene Expression Omnibus (GEO) is a public functional genomics database maintained by the National Center for Biotechnology Information. The single-cell RNA-sequencing dataset GSE173193 was obtained from GEO and included two preeclampsia samples and two control samples. The bulk transcriptomic dataset GSE74341 was also obtained from GEO and comprised expression profiles from 25 placental samples, including 15 preeclampsia samples and 10 control samples. The two datasets contained independent participants, were generated using different platforms, and did not provide harmonized clinical information. Detailed clinical metadata, including gestational age at sampling or delivery, early- or late-onset disease, disease severity, mode of delivery, labor status, fetal sex, and maternal comorbidities, were not consistently available. Therefore, these potentially important clinical variables could not be included as covariates or adjusted for in the subsequent analyses. Accordingly, the two datasets were analyzed separately: GSE173193 was used to explore cell-type heterogeneity and candidate regulatory patterns, whereas GSE74341 was used to provide complementary association-level evidence at the bulk-transcriptome level. The findings across the two datasets were therefore interpreted as complementary associations rather than as results of direct data integration or cross-dataset validation.

### Single-cell data quality control

Single-cell expression matrices were processed separately for each sample using the Seurat package. Cells expressing fewer than 200 genes were excluded. Additional quality-control filtering was performed based on the number of detected genes (nFeature_RNA), total UMI counts (nCount_RNA), mitochondrial transcript proportion (percent.mt), and ribosomal transcript proportion (percent.ribo). For each sample, the median and median absolute deviation of each quality-control metric were calculated independently, and cells with values deviating by more than three MADs from the corresponding sample median were removed. Thus, sample-specific rather than universal absolute thresholds were applied across the four samples. Doublets were subsequently identified and removed separately from each sample using DoubletFinder (version 2.0.4). After quality-control filtering and doublet removal, a total of 22,256 cells were retained for downstream analysis.

### Single-cell data dimensionality reduction, clustering and annotation

The retained cells were normalized using the Seurat LogNormalize method with a scale factor of 10,000. Cell-cycle scores were calculated using CellCycleScoring, and 2,000 highly variable genes were selected using FindVariableFeatures. ScaleData was applied while accounting for mitochondrial transcript proportion, ribosomal transcript proportion, and cell-cycle scores. Principal component analysis was performed using RunPCA, and the first 20 principal components were retained for downstream analysis. Harmony was applied using individual sample identity as the batch variable. To evaluate sample-associated variation and the effect of Harmony correction, UMAP embeddings were generated both before correction using the uncorrected PCA dimensions and after correction using the Harmony embeddings. The resulting UMAPs were visualized separately by individual sample and study group (Supplementary Figure S2). A shared nearest-neighbor graph was constructed using FindNeighbors with dimensions 1–20 and k.param=20. Clusters were identified using FindClusters with a resolution of 0.2, producing 14 transcriptionally distinct clusters.

The resolution of 0.2 was selected because it yielded stable and biologically interpretable clusters without excessive fragmentation of the major placental cell populations. The 14 clusters were assigned to eight major cell types based on canonical marker-gene expression, SingleR annotation, the CellMarker and PanglaoDB databases, and relevant literature. The cluster-level marker genes and final cell-type assignments are provided in Supplementary Table S1. Clusters exhibiting mixed or potentially ambiguous marker expression were explicitly indicated in the table and interpreted cautiously.

### Cell communication

CellChat is a tool designed to quantitatively infer and analyze intercellular communication networks using single-cell data. It employs network analysis and pattern recognition to predict the primary signal inputs and outputs of cells and to understand how these cells and signals coordinate their functions. For our analysis, we utilized standardized single-cell expression profiles as input data, along with cell subtypes identified from single-cell analysis. We examined cell-related interactions and quantified the interaction closeness by assessing the strength (weights) and frequency of interactions between cells. This approach was used to infer the relative number and strength of ligand–receptor communication events among the annotated cell populations. The resulting networks were interpreted as computationally inferred interaction patterns rather than direct measurements of cellular communication or disease-associated regulatory effects.

### Gene set enrichment analysis

The irGSEA package facilitated gene set enrichment analysis on single-cell RNA sequencing data. It incorporates multiple enrichment analysis methods, including AUCell, UCell, singscore, ssGSEA, JASMINE, and Viper, which are based on individual cell expression levels. The package employs the robust rank aggregation algorithm (RRA) to assess differential analysis results, identifying differential gene sets with consistent enrichment levels across these methods.

### SCENIC analysis

SCENIC (single-cell regulatory network inference and clustering) is a method that utilizes co-expression and motif analysis to reconstruct gene regulatory networks and identify cell states from single-cell transcriptome data. Initially, SCENIC employs GENIE3 to identify gene sets co-expressed with transcription factors. Subsequently, it conducts motif enrichment analysis on each co-expression module, retaining significantly enriched motifs, which are then annotated with transcription factors (TFs) using a database. The annotations are categorized into high and low confidence levels: high-confidence results include TFs directly annotated by the database or inferred through homologous genes, while low-confidence results involve TFs annotated via motif sequence similarity. The retained motifs are used to score genes within the co-expression module, identifying those with significantly high scores, indicating motifs are proximal to the transcription start sites (TSS) of these genes. Genes lacking high motif scores are removed from the module, and the remaining gene sets are termed regulatory units, or regulons. Each inferred regulon consists of a transcription factor and its computationally predicted target genes. A higher score indicates higher inferred regulon activity within the analyzed dataset. The resulting activity matrix facilitates the identification of cell types and states. Because transcription factors with low or sparse expression may produce less stable regulon predictions, the EVX1 regulon was interpreted cautiously as a computationally predicted candidate rather than a confirmed regulatory program. The low transcript detection of EVX1 and the limited number of biological samples were considered important sources of uncertainty in interpreting the SCENIC result.

### CytoTRACE analysis of relative transcriptional-state ordering

CytoTRACE was used to estimate relative transcriptional-state ordering based primarily on the number and diversity of genes detected in individual cells. Higher CytoTRACE scores were interpreted as reflecting relatively greater transcriptional complexity within the analyzed dataset. Because the method does not directly measure self-renewal capacity, developmental potency, or lineage relationships, the resulting scores were not interpreted as evidence of stemness or genuine differentiation potential.

### Computational pseudotime analysis

Monocle was used to arrange macrophage/monocyte cells along a computationally inferred pseudotime trajectory based on similarities in their transcriptional profiles. The analysis was used to visualize continuous transcriptional variation and genes associated with pseudotime ordering. Because the data were cross-sectional, the inferred trajectory was not interpreted as a directly observed temporal process, developmental lineage, or direction of cell differentiation.

### Immune infiltration analysis

The CIBERSORT algorithm was applied to the bulk transcriptomic dataset GSE74341 using the LM22 signature matrix to computationally estimate the relative fractions of 22 immune-cell categories. Because LM22 was developed from circulating blood leukocytes, it may not fully represent specialized placenta- or decidua-resident immune populations, including Hofbauer macrophages and decidual NK cells. Therefore, the CIBERSORT-derived fractions were interpreted as approximate computational estimates rather than direct measurements of placental immune-cell composition. We subsequently assessed the associations between EVX1/KLF9 expression and these CIBERSORT-estimated immune-cell fractions.

### GSEA

Samples were divided into high- and low-expression groups separately for each candidate gene using the median normalized expression value across all samples in GSE74341 as the cutoff. For EVX1, samples with normalized expression values greater than or equal to the median were assigned to the high-expression group, whereas those with values below the median were assigned to the low-expression group. Gene Set Enrichment Analysis (GSEA) was subsequently performed to compare pathway-level expression patterns between the two groups. Version 7.0 gene sets obtained from the Molecular Signatures Database (MSigDB) were used as the reference gene sets. Gene sets with an adjusted p value <0.05 were considered significantly enriched and were ranked according to their normalized enrichment scores. The resulting enrichment patterns were interpreted as associations with EVX1 expression rather than evidence that EVX1 directly regulates these pathways.

### GSVA

Gene set variation analysis (GSVA) is a non-parametric, unsupervised method used to assess the enrichment of transcriptome gene sets. By transforming gene-level changes into pathway-level alterations, GSVA provides comprehensive scores for gene sets, thereby determining the biological function of samples. In this study, gene sets were downloaded from the Molecular Signatures Database, and the GSVA algorithm was employed to score each gene set comprehensively. This approach evaluated potential changes in biological functions across different samples.

### Statistical analysis

Statistical analyses were conducted using R (version 4.3.0). Differences in EVX1 and KLF9 expression between the preeclampsia and control groups in GSE74341 were assessed using the Wilcoxon rank-sum test. Receiver operating characteristic curves were generated to descriptively evaluate the ability of each gene to distinguish the two groups within this dataset, and the corresponding areas under the curve were calculated. These analyses were considered complementary association-level assessments rather than independent validation. A two-sided p value <0.05 was considered statistically significant.

### Ethical approval

This study used publicly available anonymized human omics datasets from the Gene Expression Omnibus (GEO). This research was reviewed and granted an exemption by the Ethics Committee of the Affiliated Hospital of Qingdao University. All analyses were performed in accordance with the Declaration of Helsinki.

## Results

### Single-cell data quality control and clustering

To ensure data quality, cells expressing fewer than 200 genes and sample-specific outliers in gene counts, UMI counts, mitochondrial transcript proportion, and ribosomal transcript proportion were removed using the three-MAD criterion described above. Doublets were subsequently excluded using DoubletFinder. A total of 22,256 cells were retained after quality-control filtering and doublet removal. The quality-control results are illustrated in the violin and scatter plots shown in Supplementary Figure S1A and B. We subsequently identified 2,000 highly variable genes (Supplementary Figure S1C), followed by scaling, principal component analysis, and Harmony correction using the first 20 principal components (Supplementary Figure S1D–F). To assess whether the observed structure was primarily driven by sample-associated technical variation, we compared UMAP embeddings before and after Harmony correction, displaying the cells by individual sample and study group (Supplementary Figure S2). After Harmony correction, cells from different samples showed improved mixing within several major transcriptional populations. Nevertheless, because the dataset contained only four individuals and sample identity was closely associated with disease group, residual batch effects could not be fully distinguished from inter-individual or disease-associated variation.

### Cell annotation

After Harmony correction and UMAP visualization, clustering at a resolution of 0.2 identified 14 transcriptionally distinct clusters (Figure 1A). This resolution was selected because it retained biologically interpretable placental cell populations without excessive subdivision. Based on canonical marker-gene expression, SingleR annotation, the CellMarker and PanglaoDB databases, and relevant literature, the 14 clusters were assigned to eight major cell types: B cells, extravillous trophoblast cells, granulocytes, macrophages/monocytes, myelocytes, syncytiotrophoblast cells, T/NK cells, and villous cytotrophoblast cells (Figure 1B). Cluster-specific marker genes and the rationale for each annotation are summarized in Supplementary Table S1, with any clusters showing mixed or ambiguous marker expression explicitly indicated. Figure 1C presents a dot plot showing the expression of representative marker genes across all 14 clusters. The corresponding cluster-level marker genes, annotation evidence, and final cell-type assignments are summarized in Supplementary Table S1. Figure 1D shows the descriptive distributions of the eight annotated cell types across individual samples and study groups.

**Figure 1:**
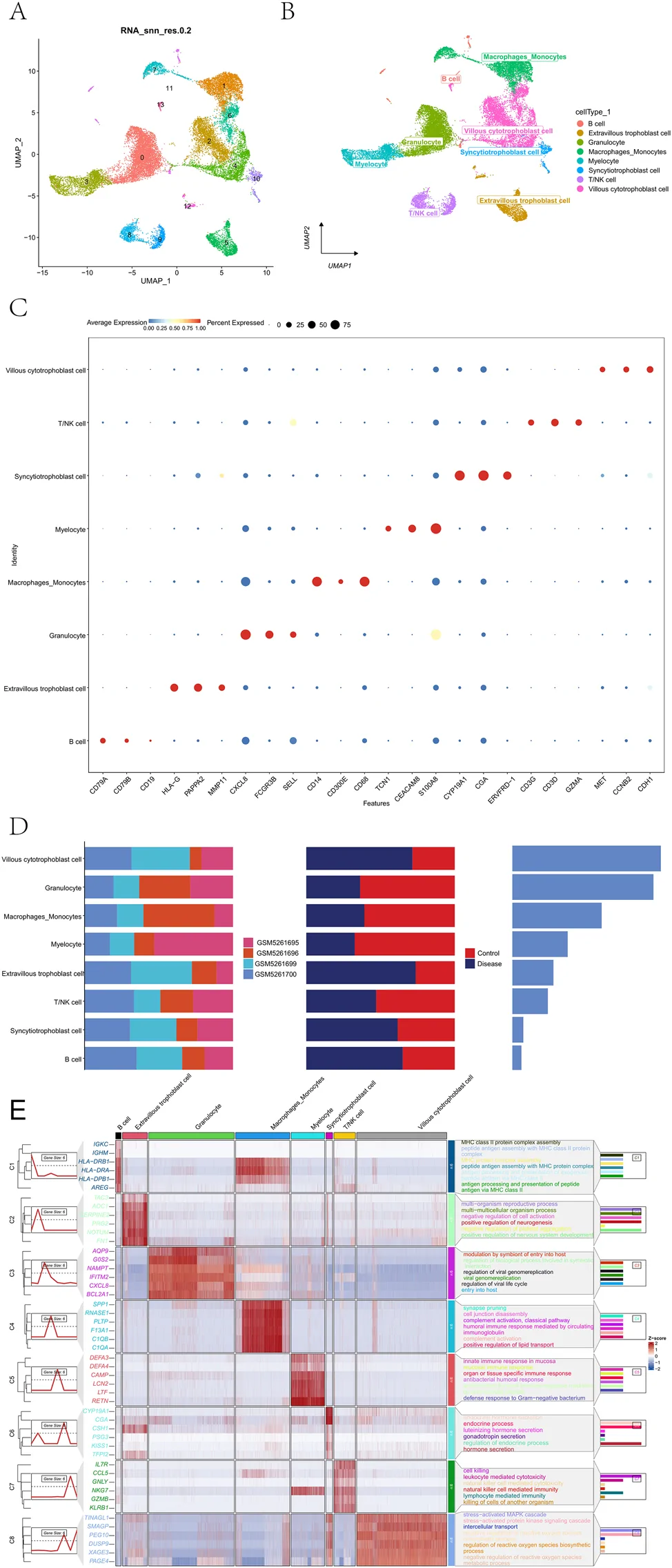
Cell annotation and subpopulation differential enrichment. (A) UMAP visualization of the 14 clusters identified using Harmony-corrected dimensions 1–20 and a clustering resolution of 0.2, (B) annotation of the 14 clusters into eight major cell types based on canonical marker genes, SingleR, CellMarker, PanglaoDB, and relevant literature: B cells, extravillous trophoblast cells, granulocytes, macrophages/monocytes, myelocytes, syncytiotrophoblast cells, T/NK cells, and villous cytotrophoblast cells, (C) dot plot showing representative marker-gene expression across the 14 clusters. Dot size indicates the percentage of cells expressing each gene, and color indicates the average normalized expression level. Detailed cluster-level annotations are provided in Supplementary Table S1, (D) descriptive distribution of the eight cell types across the individual samples and study groups and (E) pathway enrichment analysis across the eight annotated cell types.

### Differential expression and pathway enrichment across annotated cell populations

The FindAllMarkers function was used to identify differentially expressed genes across the annotated cell populations. The six genes with the highest average log2 fold changes for each population were selected for visualization. For enrichment analysis, ClusterGVis and org.Hs.eg.db were utilized, while visCluster facilitated the visualization of the heat map (Figure 1E). The analysis revealed that Macrophages_Monocytes genes were predominantly enriched in signaling pathways like synapse pruning and cell junction disassembly.

### Cell communication

We employed the software package CellChat to examine the ligand-receptor interactions within the single-cell expression profiles of the two groups. Our analysis revealed intricate interaction networks among the cell subtypes (Figure 2A and B). Within the analyzed dataset, Macrophages Monocytes (macrophages/monocytes) exhibited the highest inferred number of communication interactions and were therefore selected for exploratory downstream analysis. Given the limited number of biological samples, these results should be interpreted as descriptive communication patterns rather than statistically validated group-level differences. Lastly, we present the ligands among various cells (Figure 2C).

**Figure 2:**
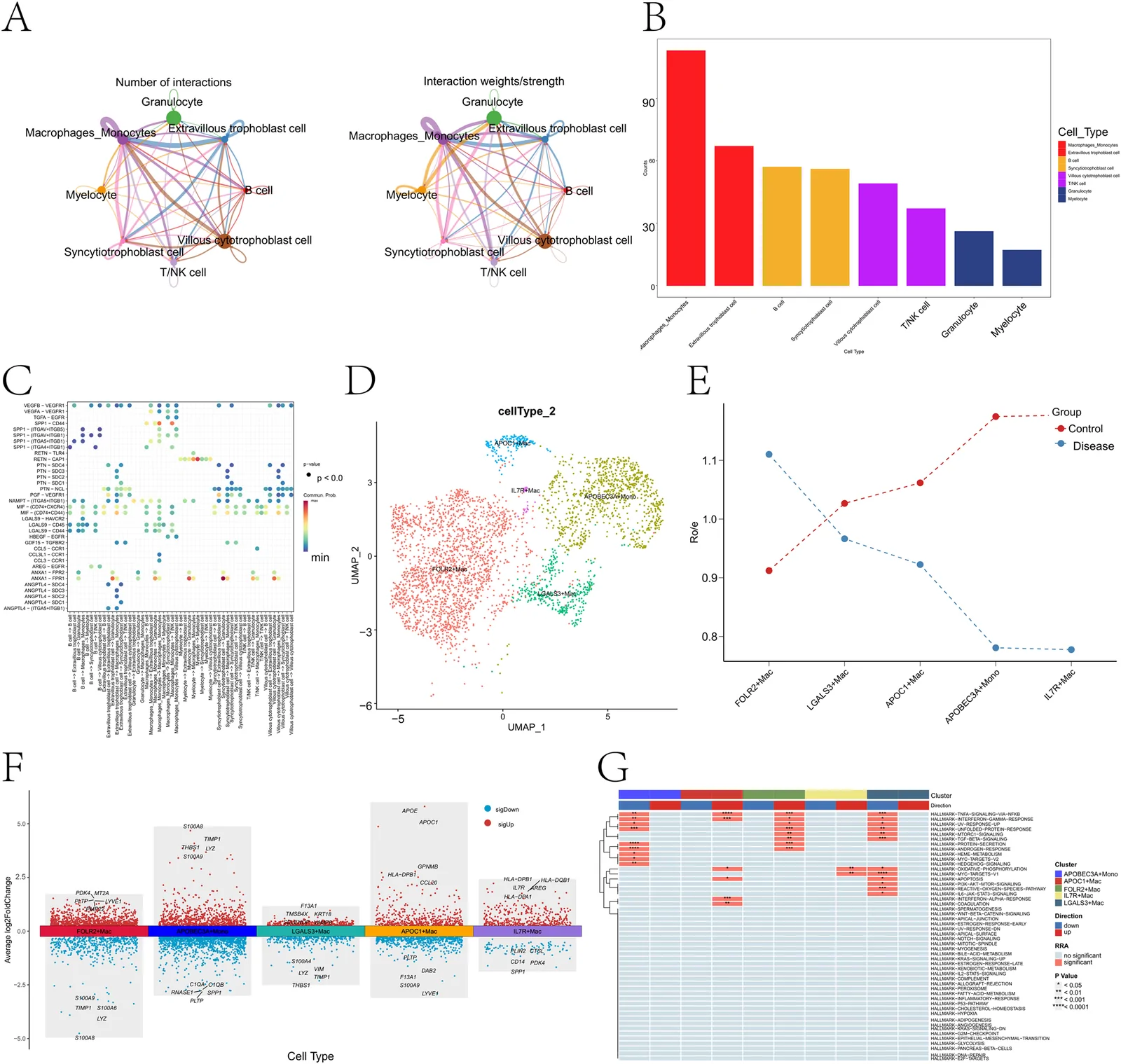
Inferred cell communication and subclassification of macrophages/monocytes. (A) The cell interaction network among the eight cell types is depicted, with edge width indicating the probability and strength of communication between the cells, (B) a comparison of the total number of interactions within the communication network across the 8 cell types, (C) dot plot showing receptor-ligand interactions between Macrophages_Monocytes and other cells, (D) Macrophages_Monocytes were subdivided into 5 subpopulations: FOLR2^+^ Mac, APOBEC3A^+^ Mono, LGALS3^+^ Mac, APOC1^+^ Mac, and IL7R^+^ Mac, (E) descriptive distribution of the five macrophage/monocyte subpopulations across the analyzed samples, (F) the volcano plot illustrates the differentially expressed genes that are either upregulated or downregulated across the five cell subtypes and (G) differential activity of HALLMARK pathways across the 5 cell subtypes.

### Subdividing Macrophages_Monocytes subgroups

Based on the inferred communication patterns, macrophages/monocytes showed the highest inferred number of communication interactions within the analyzed dataset and were therefore selected for exploratory subclustering. We subdivided the Macrophages_Monocytes population into five distinct subgroups: FOLR2^+^ Mac, APOBEC3A^+^ Mono, LGALS3^+^ Mac, APOC1^+^ Mac, and IL7R^+^ Mac (Figure 2D). The distribution of these cell types varied across different groups (Figure 2E), with FOLR2^+^ Mac showing a relatively higher observed proportion in the preeclampsia samples included in this dataset. Given the small number of individuals, this pattern was considered descriptive and was not interpreted as statistically validated enrichment in preeclampsia. The five subgroups displayed significant gene expression changes, both up-regulated and down-regulated (Figure 2F).

### Gene enrichment analysis

The irGSEA package facilitated gene set enrichment analysis on Macrophages_Monocytes single-cell RNA sequencing data. Enrichment scores were calculated using AUCell, UCell, singscore, and ssGSEA. Results from the four methods were combined using robust rank aggregation to identify gene sets showing consistent differences in inferred pathway activity across macrophage/monocyte subpopulations. The top 50 differential gene sets were visualized using heatmaps, illustrating differences in pathway-level transcriptional signatures across the five subpopulations (Figure 2G).

### SCENIC analysis identifies candidate regulons in macrophage/monocyte subpopulations

We conducted SCENIC analysis on the Macrophages_Monocytes subpopulation, focusing on five subpopulations: FOLR2^+^ Mac, APOBEC3A^+^ Mono, LGALS3^+^ Mac, APOC1^+^ Mac, and IL7R^+^ Mac, along with disease controls. The analysis primarily examined the specificity scores of significant regulators (Figure 3A–G). A higher score on the vertical axis indicates stronger regulator specificity within the cell, while the horizontal axis ranks these regulators by specificity score, with higher rankings denoting greater specificity. The heat map (Figure 3H) illustrates the Connection Specificity Index (CSI), which measures the connection specificity between different modules. Each square represents the CSI value for a module pair, with darker colors indicating higher specificity (high CSI value) and lighter colors indicating lower specificity (low CSI value). Figure 3I presents the average activity score map for six modules (Module1 to Module6), revealing that FOLR2^+^ Mac exhibits the highest average activity in M1. Subsequently, Figure 3J–O displays the ranking of gene contributions to overall variance within each regulatory network module. The horizontal axis ranks genes by variance, while the vertical axis shows each gene’s variance proportion relative to the total variance. Colored points denote genes within the module, whereas gray points represent genes in other modules. Higher-ranked genes may exert stronger regulatory effects within the network, aiding in identifying key transcription factors contributing to expression variation in specific modules. Finally, we examined the overlap among regulators associated with Module M1, the FOLR2^+^ Mac subpopulation, and disease status. This analysis identified EVX1 and KLF9 as computationally predicted candidate regulators (Figure 3P). Given the limited number of biological samples and the computational nature of SCENIC, these findings were considered exploratory rather than evidence of validated regulatory activity. EVX1 was also noted to have low and sparse transcript detection, further limiting confidence in its inferred regulon.

**Figure 3:**
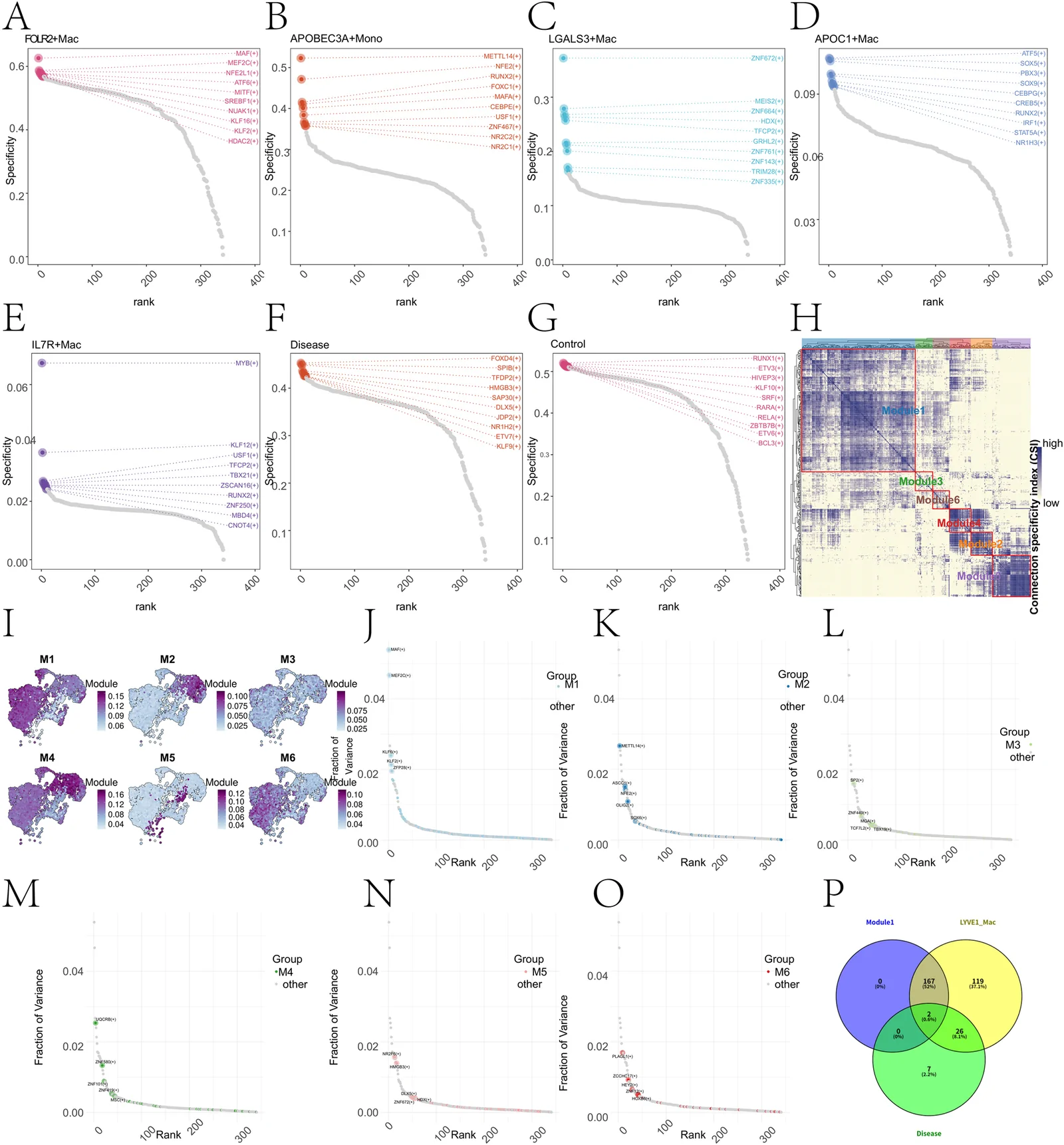
SCENIC analysis and visualization of inferred regulon activity. (A–E) Rankings of regulon specificity across FOLR2^+^ Mac, APOBEC3A^+^Mono, LGALS3^+^ Mac, APOC1^+^ Mac, and IL7R^+^ Mac subpopulations, (F–G) rankings of regulon specificity in preeclampsia and control cells, (H) Connection Specificity Index heatmap showing relationships among inferred regulon modules, (I) UMAP visualization of the inferred activity scores of modules M1–M6, (J–O) rankings of regulators contributing to variation within modules M1–M6 and (P) Venn diagram showing the overlap among module M1-associated regulators, FOLR2^+^ Mac-associated regulators, and disease-associated regulators.

In the complementary bulk transcriptomic dataset GSE74341, EVX1 expression was higher, whereas KLF9 expression was lower, in the preeclampsia group than in the control group. ROC analyses yielded AUC values of 0.807 for EVX1 and 0.800 for KLF9. Because GSE74341 formed part of the present multi-dataset analysis and no external cohort was available, these findings represent within-dataset association and discrimination analyses rather than independent validation (Supplementary Figure S3).

### CytoTRACE and pseudo-time analysis

CytoTRACE assesses relative transcriptional-state differences among individual cells based on their gene expression profiles. A higher score may reflect greater transcriptional complexity or a larger number of detected genes. Our findings revealed that the FOLR2^+^ Mac subpopulation exhibited a relatively high CytoTRACE score within the analyzed dataset (Figure 4A). Given the limited number of biological samples, this ranking should be interpreted as exploratory and should not be considered a statistically validated group-level difference or evidence of genuine stemness or developmental potential. Additionally, the relative positions of various cell types along the computationally inferred transcriptional-state trajectory were mapped, highlighting transcriptional heterogeneity among subpopulations (Figure 4B). We then focused on the Macrophages_Monocytes subpopulation for pseudo-time analysis. Initially, we calculated cell similarities to construct the cell differentiation trajectory. This trajectory visualization, based on pseudo-time, illustrates the cell development process, enabling the study of cell differentiation and gene expression patterns at different time points. Pseudotime values (calculated by Monocle based on gene expression data), states (distinguished by path branches), and images of cells colored by different groups were generated (Figure 5A–C). We also identified a set of genes with significant changes along the pseudo-time for visualization. The horizontal axis represents the pseudo-time value, while the vertical axis shows the selected genes. These genes were categorized into three clusters by default. Notably, genes like MT2A were expressed early in the differentiation trajectory, whereas genes such as CD48 appeared later (Figure 5D).

**Figure 4:**
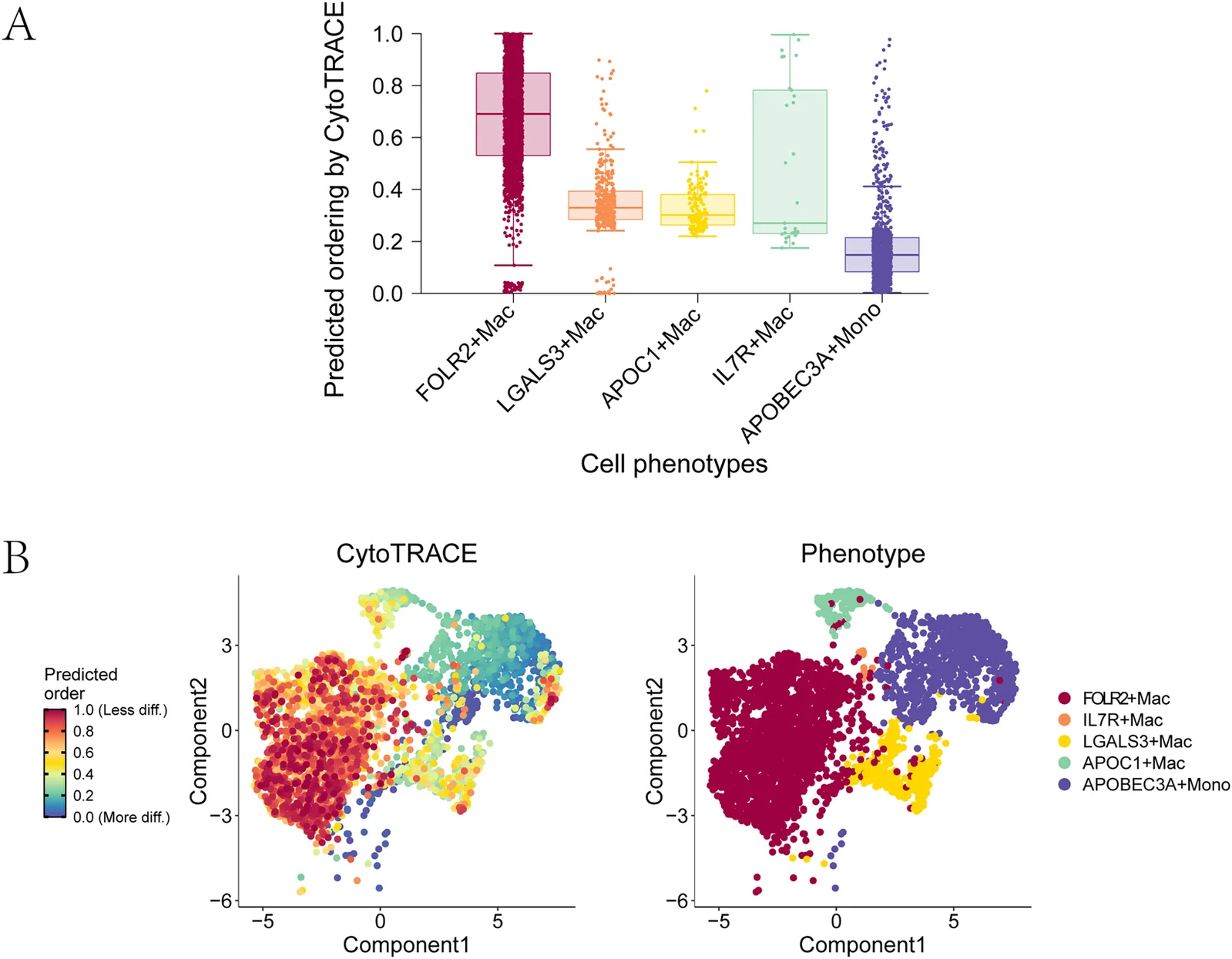
Relative transcriptional-state ordering of macrophage and monocyte subpopulations inferred by CytoTRACE. (A) Distribution of CytoTRACE scores across the five macrophage/monocyte subpopulations. Higher scores indicate higher relative CytoTRACE ordering within the analyzed dataset and should not be interpreted as direct evidence of stemness or developmental potential, (B) visualization of CytoTRACE scores and subpopulation identities in reduced-dimensional space.

**Figure 5:**
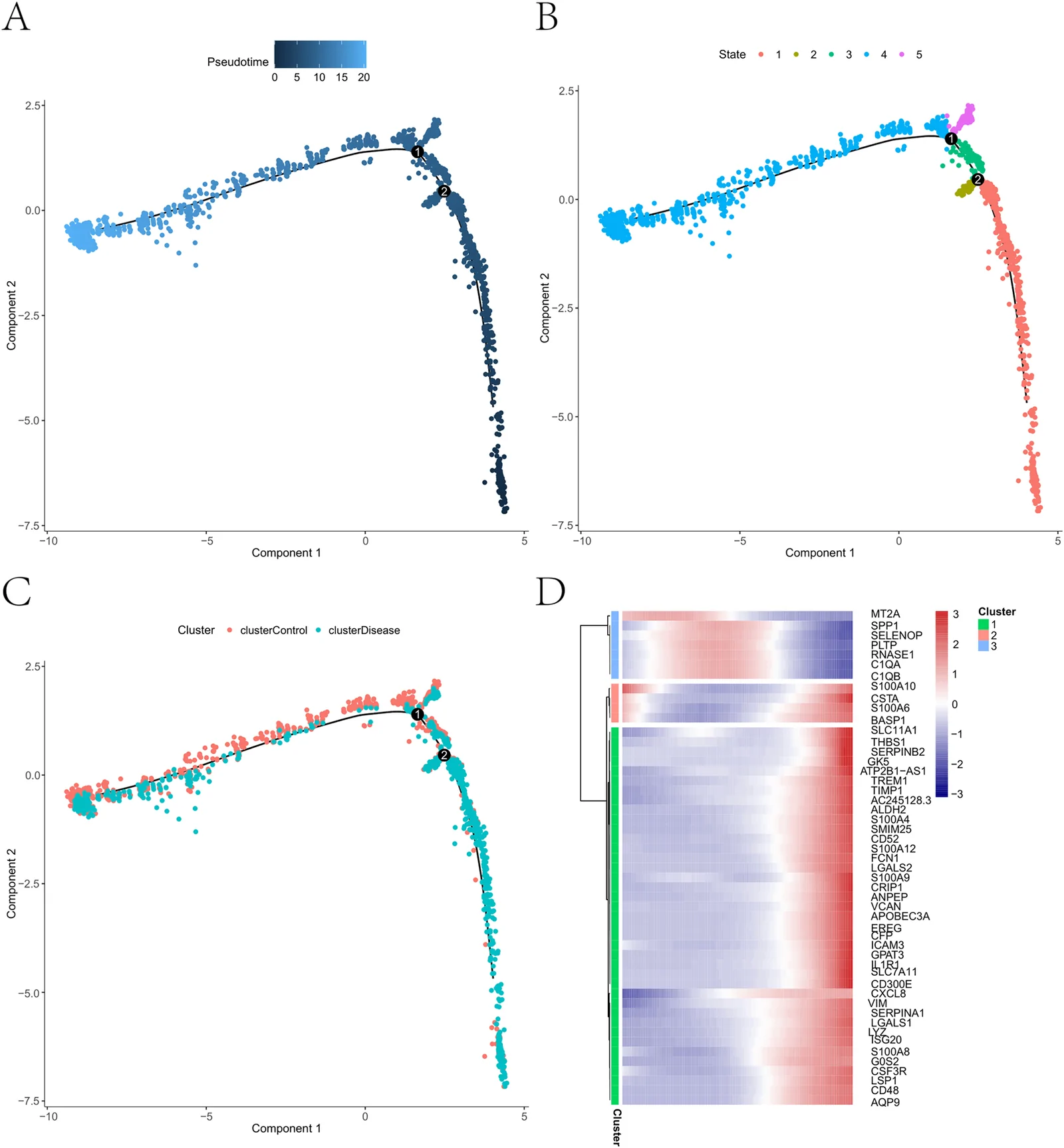
Computational pseudotime ordering of macrophage and monocyte subpopulations. (A) Cells colored by computational pseudotime, (B) cells colored by inferred pseudotime state, (C) distribution of control and preeclampsia cells along the inferred trajectory and (D) gene-expression patterns associated with pseudotime ordering. The trajectory represents computational transcriptional ordering and does not establish a temporal lineage relationship.

### Immune infiltration analysis

CIBERSORT was used to estimate the relative fractions of the 22 LM22 immune-cell categories in the bulk transcriptomic samples, and correlations among these estimated fractions are shown in Figure 6A and B. Compared with the control group, the preeclampsia group showed higher estimated fractions of activated dendritic cells and resting mast cells and a lower estimated fraction of activated CD4^+^ memory T cells (Figure 6C). EVX1 expression was positively associated with the estimated fractions of naive B cells, activated dendritic cells, and resting mast cells and negatively associated with those of memory B cells and activated CD4^+^ memory T cells. KLF9 expression was positively associated with the estimated M0 macrophage fraction and negatively associated with the estimated fractions of naive B cells and eosinophils (Figure 6D). Because LM22 was derived from circulating blood leukocytes, these categories may not accurately represent specialized placenta- or decidua-resident immune populations. Accordingly, the observed differences should be interpreted as approximate computational estimates rather than directly measured changes in placental immune-cell abundance. In addition, because gestational age and other relevant clinical variables were unavailable, these differences cannot be attributed exclusively to preeclampsia. Associations between candidate regulator expression and chemokines, receptors, MHC molecules, immunoinhibitors, and immunostimulators are shown in Figure 7A–E.

**Figure 6:**
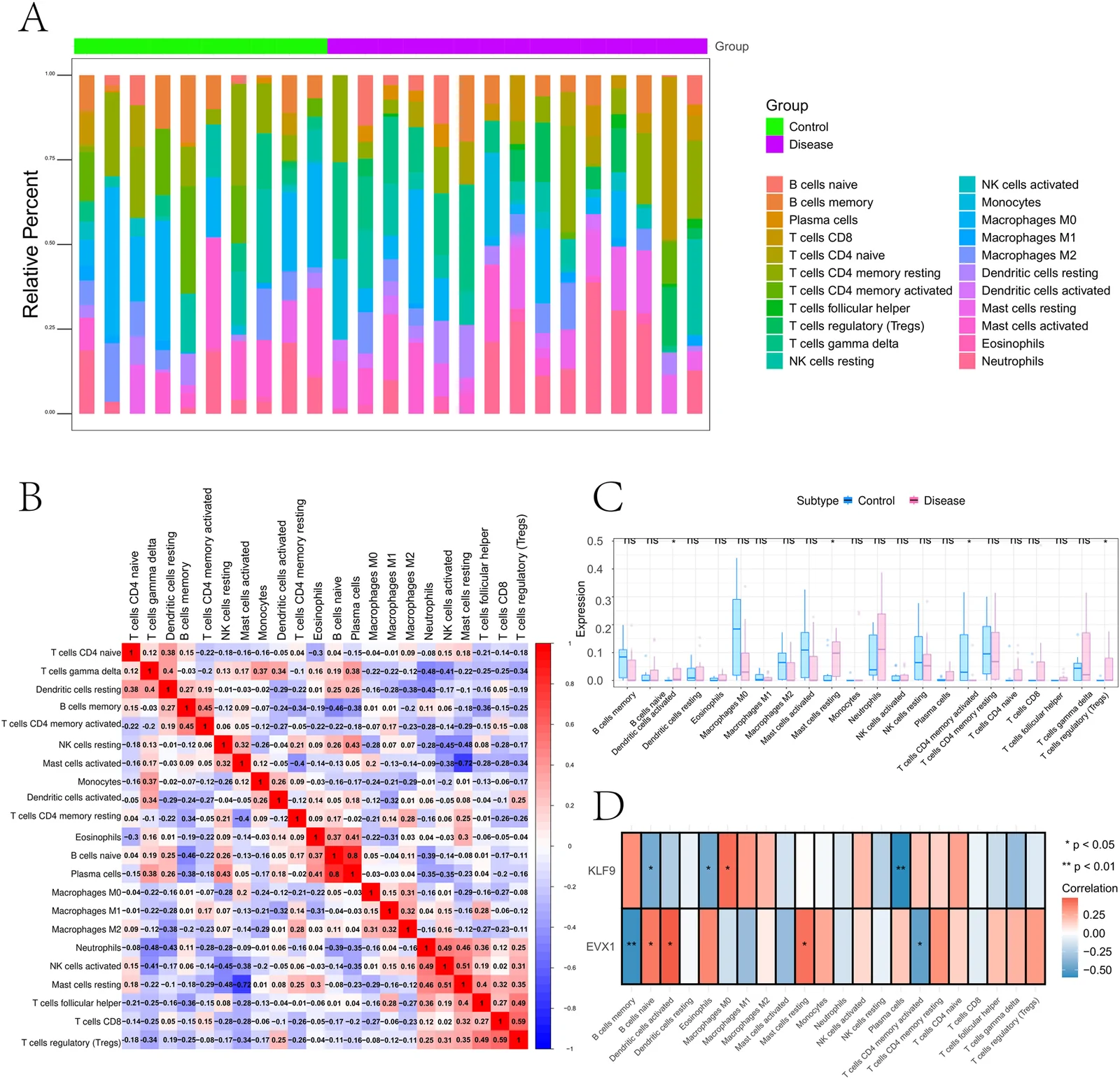
CIBERSORT-estimated immune-cell fractions in the bulk transcriptomic dataset. (A) CIBERSORT-estimated relative fractions of the 22 LM22 immune-cell categories in the bulk transcriptomic samples, (B) correlations among the CIBERSORT-estimated immune-cell fractions, with blue representing negative correlations and red indicating positive correlations, (C) comparison of the CIBERSORT-estimated immune-cell fractions between control and preeclampsia samples and (D) associations between candidate regulator expression and CIBERSORT-estimated immune-cell fractions.

**Figure 7:**
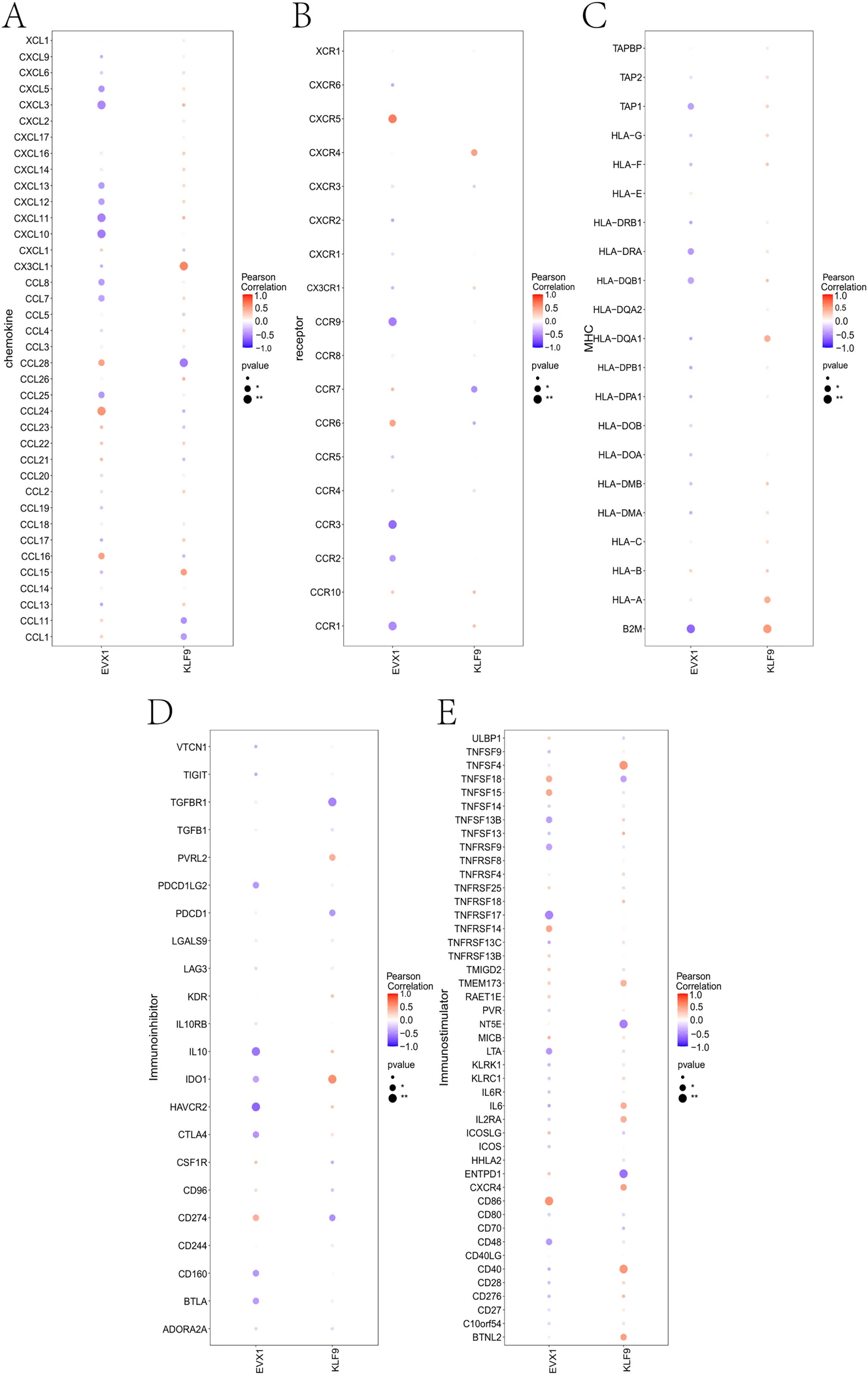
Associations between candidate regulator expression and immune-related factors. (A) Chemokines, (B) chemokine receptors and other immune receptors, (C) MHC-related molecules, (D) immunoinhibitory factors and (E) immunostimulatory factors. Dot color indicates the Pearson correlation coefficient, and dot size indicates the corresponding p value.

### GSEA and GSVA

We examined pathway-level transcriptional signatures associated with EVX1 and KLF9 expression. GSEA identified associations between the EVX1 expression groups and oxidative phosphorylation, retrograde endocannabinoid signaling, and thermogenesis-related signatures (Figure 8A). The KLF9 expression groups differed in ascorbate and aldarate metabolism, pantothenate and CoA biosynthesis, and protein digestion and absorption-related signatures (Figure 8B). GSVA further identified differences in coagulation-related scores between the EVX1 expression groups and differences in IL6/JAK/STAT3-and PI3K/AKT/mTOR-related scores between the KLF9 expression groups (Figure 8C and D). These findings represent pathway-level associations within GSE74341 and do not demonstrate direct regulation of these pathways by EVX1 or KLF9.

**Figure 8:**
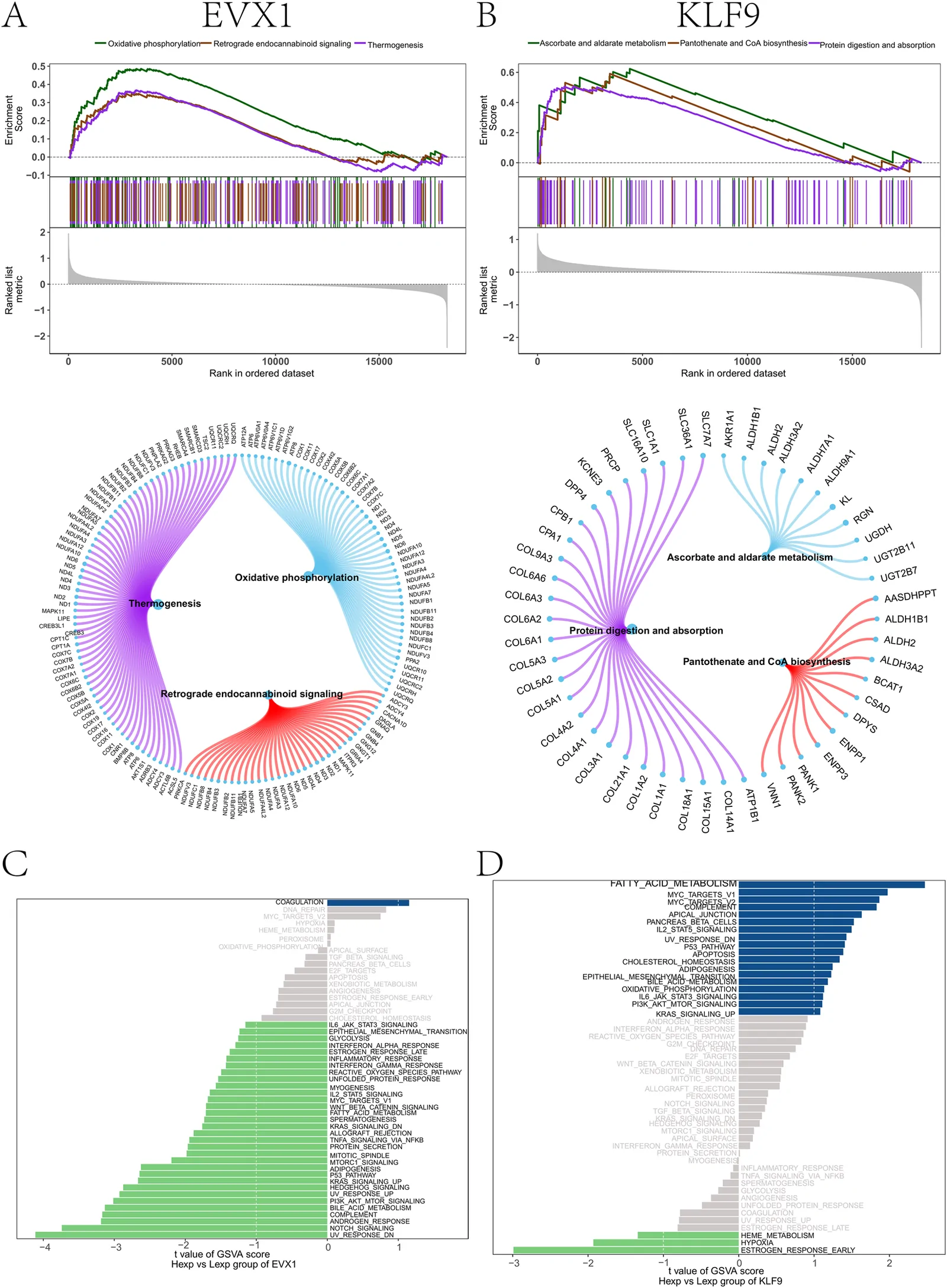
Pathway-level signatures associated with EVX1 and KLF9 expression. (A) GSEA of pathway signatures associated with EVX1 expression, (B) GSEA of pathway signatures associated with KLF9 expression, (C) GSVA comparison between EVX1-high and EVX1-low samples, (D) GSVA comparison between KLF9-high and KLF9-low samples. These analyses indicate statistical associations and do not establish direct pathway regulation.

### Correlation between key regulators and immune metabolism pathways

AUCell was used to calculate immune- and metabolism-related pathway scores within the macrophage/monocyte compartment. Differences in pathway scores associated with EVX1 and KLF9 were visualized using a bubble plot. EVX1 and KLF9 showed associations with several pathway-level signatures, including complement-and androgen response-related signatures (Supplementary Figure S4). These results represent computational associations and do not establish direct pathway regulation.

## Discussion

Preeclampsia (PE) remains a significant contributor to increased maternal and perinatal mortality rates. Although various therapeutic approaches exist, no completely effective cure has been established [26]. Current management strategies primarily focus on controlling hypertension, preventing convulsions, and ensuring timely termination of pregnancy. Antihypertensive agents, such as labetalol and nifedipine, are commonly utilized to alleviate symptoms, while magnesium sulfate serves as the first-line medication for preventing and treating eclamptic seizures [27]. However, the only definitive treatment is the delivery of the fetus and placenta. Preventive measures, including low-dose aspirin and calcium supplementation, have demonstrated efficacy in high-risk populations [28], 29]. These interventions depend on early identification and proactive management [30], and their therapeutic effects are limited for women who have already developed preeclampsia [31]. Further investigation of the cellular and molecular alterations associated with PE may improve understanding of its biological heterogeneity and support the generation of hypotheses for future mechanistic studies [32].

The present study was designed to identify candidate cellular and transcriptional patterns associated with preeclampsia through computational analyses of publicly available datasets. Accordingly, the identified macrophage/monocyte subpopulations and candidate regulators should not be regarded as established pathogenic drivers, validated biomarkers, or therapeutic targets. Their potential biological and clinical relevance requires confirmation in independent placental cohorts and through tissue-based and functional experiments.

Preeclampsia is a multifactorial disease characterized by various pathological mechanisms and signaling pathways. Many researchers propose the “two-stage” theory of its pathogenesis, which involves impaired trophoblast invasion and spiral artery remodeling during early pregnancy, accompanied by dysregulated immune responses, followed by systemic inflammatory activation in the mother during late gestation [33]. Patients with preeclampsia exhibit excessive activation of inflammatory and immune responses, occurring both locally at the maternal-fetal interface and systemically throughout the body [34], [35], [36]. In recent years, immune cell infiltration has been recognized as a critical factor in the pathogenesis and progression of preeclampsia (PE) [37]. Investigations into the immunological mechanisms underlying preeclampsia have demonstrated that aberrant immune responses, particularly an immune imbalance at the maternal-fetal interface, represent a key pathological mechanism in PE [38], [39], [40]. During normal pregnancy, the placental immune microenvironment maintains a state of immune tolerance; however, in patients with PE, this balance is disrupted, leading to abnormal infiltration and activation of immune cells. Specifically, macrophages, T cells, and dendritic cells exhibit dysregulated proliferation and functional impairments in PE. Through the secretion of cytokines and chemokines, these immune cells exacerbate inflammatory responses and vascular dysfunction, thereby promoting the development and progression of PE [22], 41], 42].

This study identified macrophage- and monocyte-associated transcriptional patterns in the analyzed preeclampsia dataset, suggesting a potential association between these cell populations and alterations in the placental immune microenvironment. CIBERSORT analysis estimated relatively higher fractions of activated dendritic cells and resting mast cells and a relatively lower fraction of activated CD4^+^ memory T cells in the preeclampsia group. However, these findings should be interpreted cautiously because CIBERSORT used the blood-derived LM22 reference matrix, which may not adequately represent specialized placental or decidual immune populations, including Hofbauer macrophages and decidual NK cells. The inferred fractions therefore represent approximate computational estimates rather than direct measurements of placental immune-cell abundance. In addition, the limited sample size and the absence of gestational-age and other clinical covariates prevent these differences from being attributed exclusively to preeclampsia.

Using complementary analyses of two independent datasets, this study identified FOLR2^+^ macrophages (FOLR2^+^ Mac) and APOBEC3A^+^ monocytes (APOBEC3A^+^ Mono) as candidate cell populations associated with preeclampsia. The single-cell dataset was used to explore cellular heterogeneity and candidate regulatory patterns, whereas the bulk dataset provided complementary association-level evidence. Because the datasets did not share participants, platforms, or harmonized clinical information, the bulk analysis should not be considered direct cross-dataset validation of the single-cell findings. FOLR2 is commonly expressed by tissue-resident macrophage populations that have been associated with anti-inflammatory, tissue-repair, and immunoregulatory phenotypes in several tissue contexts. FOLR2^+^ macrophages have also been investigated as prognostic or therapeutic targets in solid tumors [43], 44]. However, findings from tumor-associated macrophages cannot be directly extrapolated to placental macrophages in preeclampsia. Similarly, APOBEC3A^+^ monocytes represent a transcriptionally distinct monocyte state involving the cytidine deaminase APOBEC3A, which has been linked to immune defense and disease-related processes [45]. The present analysis does not establish the functional roles of either subpopulation in placental immune tolerance, angiogenesis, or disease progression.

Using SCENIC analysis, we identified EVX1 and KLF9 as core transcriptional regulators with significant roles in the immune microenvironment. EVX1 primarily enriched in signaling pathways associated with oxidative phosphorylation and heat shock response, indicating its potential role in regulating energy metabolism processes that influence placental function and maternal-fetal immune responses. Concurrently, KLF9 exhibited substantial enrichment in the IL6/JAK/STAT3 and PI3K/AKT/mTOR signaling pathways, which are well-established in immune responses, inflammation, and angiogenesis [46], [47], [48], [49]. The activation of KLF9 within these pathways may further modulate immune cell differentiation and function, thereby contributing to the pathogenesis and progression of preeclampsia (PE).

Even-Skipped Homeobox 1 (EVX1) is a highly conserved homeobox transcription factor that plays a critical role in embryonic development and morphogenesis; however, its expression is typically silenced in adulthood. The abnormal reactivation of EVX1 is closely associated with the initiation and progression of various malignant tumors [50]. In the present dataset, the low and sparse transcript detection of EVX1, together with the limited number of biological samples, reduces confidence in the stability of its SCENIC-inferred regulon. EVX1 should therefore be considered an exploratory computational candidate rather than a confirmed macrophage regulator. KLF9, a member of the Krüppel-like factor (KLF) family, primarily regulates gene expression and participates in numerous critical processes, including cell proliferation, apoptosis, embryonic development, metabolic regulation, and inflammatory responses. The functional inactivation of KLF9 represents a key event in the pathogenesis of many cancers and other significant diseases [51], 52]. However, the current analyses do not establish a functional role for KLF9 in placental immune cells. The expression comparisons and ROC analyses in GSE74341 provide within-dataset association and discrimination evidence rather than independent validation.

GSEA and GSVA identified distinct immune-and metabolism-related pathway signatures associated with EVX1 and KLF9 expression. These computational associations generate hypotheses regarding potential immunometabolic alterations in preeclampsia but do not establish direct regulatory mechanisms, diagnostic value, or therapeutic relevance. Independent tissue-level expression analyses and functional perturbation experiments are required before either gene can be considered a biomarker or therapeutic target.

Several limitations should be acknowledged. First, the single-cell dataset included only two preeclampsia and two control individuals. Although 22,256 cells were retained after quality control, individual cells do not constitute independent biological replicates. Therefore, the observed differences in cell proportions, inferred communication networks, pathway scores, regulon activity, and CytoTRACE ordering may be influenced by inter-individual variability and should be considered exploratory and hypothesis-generating. Second, the two datasets contained independent participants and lacked harmonized clinical information. Detailed metadata, including gestational age at sampling or delivery, early- or late-onset disease, disease severity, mode of delivery, labor status, fetal sex, and maternal comorbidities, were not consistently available. Consequently, these potentially important confounding factors could not be adjusted for, and some observed differences may reflect gestational or clinical heterogeneity rather than preeclampsia alone. Third, although Harmony correction improved the mixing of cells from different samples within several major transcriptional populations, sample identity was closely associated with disease group in this four-sample dataset. Residual technical effects therefore could not be fully separated from inter-individual or disease-associated variation. Fourth, the blood-derived LM22 reference used for CIBERSORT may not adequately represent placenta- or decidua-resident immune populations, and the estimated immune-cell fractions should be interpreted as approximate computational estimates. Fifth, no independent cohort or tissue-based experimental validation was available for the FOLR2^+^ macrophage and APOBEC3A^+^ monocyte subpopulations or for EVX1 and KLF9. No qPCR, Western blotting, immunohistochemistry, immunofluorescence, or functional perturbation experiments were performed. Moreover, the low transcript detection of EVX1 may reduce the robustness of its SCENIC-derived regulon. Finally, CytoTRACE and pseudotime analyses provide relative computational transcriptional ordering and should not be interpreted as direct evidence of stemness, developmental potential, temporal progression, or lineage relationships. Larger, clinically well-characterized independent placental cohorts and functional experiments are required to validate these observations.

## Conclusions

This study performed complementary analyses of independent single-cell and bulk transcriptomic datasets to explore cellular and transcriptional patterns associated with preeclampsia. Within the analyzed single-cell dataset, macrophages/monocytes showed relatively high inferred communication activity, while FOLR2^+^ macrophages and APOBEC3A^+^ monocytes were identified as candidate subpopulations of interest. SCENIC analysis further suggested EVX1 and KLF9 as computationally predicted candidate regulators. These findings represent exploratory associations rather than established disease mechanisms. Because of the limited number of biological samples and the absence of detailed clinical information, the potential effects of gestational age, inter-individual variability, and other clinical factors cannot be excluded. Larger, clinically well-characterized independent cohorts and experimental studies are required to validate these observations.

## Supplementary Material

Supplementary Material

Supplementary Material

## References

[1] Kuklina EV, Ayala C, Callaghan WM (2009). Hypertensive disorders and severe obstetric morbidity in the United States. Obstet Gynecol.

[2] Khan KS, Wojdyla D, Say L, Gülmezoglu AM, Van Look PF (2006). WHO analysis of causes of maternal death: a systematic review. Lancet.

[3] Gestational Hypertension and Preeclampsia: ACOG Practice Bulletin (2020). Gestational Hypertension and Preeclampsia: ACOG Practice Bulletin, Number 222. Obstet Gynecol.

[4] Mol BWJ, Roberts CT, Thangaratinam S, Magee LA, de Groot CJM, Hofmeyr GJ (2016). Pre-eclampsia. Lancet.

[5] Dimitriadis E, Rolnik DL, Zhou W, Estrada-Gutierrez G, Koga K, Francisco RPV (2023). Pre-eclampsia. Nat Rev Dis Primers.

[6] Liu XH, Wang XD, He GL (2015). Research status of the pathogenesis of pre-eclampsia. Sichuan Da Xue Xue Bao Yi Xue Ban.

[7] Karumanchi SA (2024). Two decades of advances in preeclampsia research: molecular mechanisms and translational studies. J Clin Invest.

[8] Wu Y, Sun T, Medina P, Narasimhan P, Stevenson DK, Von Versen-Höynck F (2024). A novel stem cell model to study preeclampsia endothelial dysfunction. Reprod Sci.

[9] Barbouti A, Varvarousis DN, Kanavaros P (2025). The role of oxidative stress-induced senescence in the pathogenesis of preeclampsia. Antioxidants (Basel).

[10] Aouache R, Biquard L, Vaiman D, Miralles F (2018). Oxidative stress in preeclampsia and placental diseases. Int J Mol Sci.

[11] Miller D, Motomura K, Galaz J, Gershater M, Lee ED, Romero R (2022). Cellular immune responses in the pathophysiology of preeclampsia. J Leukoc Biol.

[12] Foo L, Tay J, Lees CC, McEniery CM, Wilkinson IB (2015). Hypertension in pregnancy: natural history and treatment options. Curr Hypertens Rep.

[13] Cox AG, Marshall SA, Palmer KR, Wallace EM (2019). Current and emerging pharmacotherapy for emergency management of preeclampsia. Expert Opin Pharmacother.

[14] Roberts JM, Himes KP (2017). Pre-eclampsia: screening and aspirin therapy for prevention of pre-eclampsia. Nat Rev Nephrol.

[15] Vendelbo JH, Thunbo M, Henriksen TB, Liew Z, Larsen A, Pedersen LH (2025). The nationwide impact of guidelines for prophylactic aspirin treatment for preeclampsia. Paediatr Perinat Epidemiol.

[16] Maeda Y, Suzuki Y, Yamamoto K, Tanimoto T (2019). Prepregnancy calcium ementation and pre-eclampsia. Lancet.

[17] Liu YH, Zhang YS, Chen JY, Wang ZJ, Liu YX, Li JQ (2023). Comparative effectiveness of prophylactic strategies for preeclampsia: a network meta-analysis of randomized controlled trials. Am J Obstet Gynecol.

[18] Poon LC, Shennan A, Hyett JA, Kapur A, Hadar E, Divakar H (2019). The International Federation of Gynecology and Obstetrics (FIGO) initiative on pre-eclampsia: a pragmatic guide for first-trimester screening and prevention. Int J Gynaecol Obstet.

[19] Vishnyakova P, Elchaninov A, Fatkhudinov T, Sukhikh G (2019). Role of the monocyte-macrophage system in normal pregnancy and preeclampsia. Int J Mol Sci.

[20] Wei Y, Su Y, Liu C, Ma X, Ling Z, Wang Y (2025). Macrophage and preeclampsia: macrophage polarization imbalance at the maternal-fetal interface. J Clin Lab Anal.

[21] Ji ZS, Jiang H, Xie Y, Wei QP, Yin XF, Ye JH (2022). Chemerin promotes the pathogenesis of preeclampsia by activating CMKLR1/p-Akt/CEBPɑ axis and inducing M1 macrophage polarization. Cell Biol Toxicol.

[22] Rong M, Yan X, Zhang H, Zhou C, Zhang C (2021). Dysfunction of decidual macrophages is a potential risk factor in the occurrence of preeclampsia. Front Immunol.

[23] Todros T, Paulesu L, Cardaropoli S, Rolfo A, Masturzo B, Ermini L (2021). Role of the macrophage migration inhibitory factor in the pathophysiology of pre-eclampsia. Int J Mol Sci.

[24] Milosevic-Stevanovic J, Krstic M, Radovic-Janosevic D, Popovic J, Tasic M, Stojnev S (2016). Number of decidual natural killer cells & macrophages in pre-eclampsia. Indian J Med Res.

[25] Carney E (2016). Altered placental macrophages in pre-eclampsia. Nat Rev Nephrol.

[26] Grotegut CA (2016). Prevention of preeclampsia. J Clin Invest.

[27] McCoy S, Baldwin K (2009). Pharmacotherapeutic options for the treatment of preeclampsia. Am J Health Syst Pharm.

[28] Roberge S, Nicolaides K, Demers S, Hyett J, Chaillet N, Bujold E (2017). The role of aspirin dose on the prevention of preeclampsia and fetal growth restriction: systematic review and meta-analysis. Am J Obstet Gynecol.

[29] Belhomme N, Doudnikoff C, Polard E, Henriot B, Isly H, Jego P (2017). Aspirin: indications and use during pregnancy. Rev Med Interne.

[30] Zhang Y, Gu X, Yang N, Xue Y, Ma L, Wang Y (2025). Prediction models for late-onset preeclampsia: a study based on logistic regression, support vector machine, and extreme gradient boosting models. Biomedicines.

[31] Bujold E (2022). Optimal screening for preeclampsia in the first trimester of pregnancy. Hypertension.

[32] Chiang YT, Seow KM, Chen KH (2024). The pathophysiological, genetic, and hormonal changes in preeclampsia: a systematic review of the molecular mechanisms. Int J Mol Sci.

[33] Matthiesen L, Berg G, Ernerudh J, Ekerfelt C, Jonsson Y, Sharma S (2005). Immunology of preeclampsia. Chem Immunol Allergy.

[34] Apicella C, Ruano CSM, Méhats C, Miralles F, Vaiman D (2019). The role of epigenetics in placental development and the etiology of preeclampsia. Int J Mol Sci.

[35] Chakraborty D, Cui W, Rosario GX, Scott RL, Dhakal P, Renaud SJ (2016). HIF-KDM3A-MMP12 regulatory circuit ensures trophoblast plasticity and placental adaptations to hypoxia. Proc Natl Acad Sci USA.

[36] Deng Z, Zhang L, Tang Q, Xu Y, Liu S, Li H (2020). Circulating levels of IFN-γ, IL-1, IL-17 and IL-22 in pre-eclampsia: a systematic review and meta-analysis. Eur J Obstet Gynecol Reprod Biol.

[37] Meng Y, Li C, Liu CX (2021). Immune cell infiltration landscape and immune marker molecular typing in preeclampsia. Bioengineered.

[38] Scholz C, Toth B, Santoso L, Kuhn C, Franz M, Mayr D (2008). Distribution and maturity of dendritic cells in diseases of insufficient placentation. Am J Reprod Immunol.

[39] Robertson SA, Green ES, Care AS, Moldenhauer LM, Prins JR, Hull ML (2019). Therapeutic potential of regulatory T cells in preeclampsia-opportunities and challenges. Front Immunol.

[40] Alijotas-Reig J, Esteve-Valverde E, Ferrer-Oliveras R, Llurba E, Gris JM (2017). Tumor necrosis factor-alpha and pregnancy: focus on biologics. An updated and comprehensive review. Clin Rev Allergy Immunol.

[41] Peng X, Chinwe Oluchi-Amaka I, Kwak-Kim J, Yang X (2025). A comprehensive review of the roles of T-cell immunity in preeclampsia. Front Immunol.

[42] Huang SJ, Chen CP, Schatz F, Rahman M, Abrahams VM, Lockwood CJ (2008). Pre-eclampsia is associated with dendritic cell recruitment into the uterine decidua. J Pathol.

[43] Jiang D, Xiao R, Bai J, Wang X, Fang Y, Liu J (2025). FOLR2(^+^) macrophages in cancer: allies or enemies. Cell Commun Signal.

[44] (2022). FOLR2^+^ macrophages are associated with T-cell infiltration and improved prognosis. Cancer Discov.

[45] Smith NJ, Reddin I, Policelli P, Oh S, Zainal N, Howes E (2025). Differentiation signals induce APOBEC3A expression via GRHL3 in squamous epithelia and squamous cell carcinoma. Embo J.

[46] Amara CS, Kami Reddy KR, Yuntao Y, Chan YS, Piyarathna DWB, Dobrolecki LE (2024). The IL6/JAK/STAT3 signaling axis is a therapeutic vulnerability in SMARCB1-deficient bladder cancer. Nat Commun.

[47] Yang H, Zhang J, Li J, Zhao F, Shen Y, Xing X (2018). Overexpression of miR-574-3p suppresses proliferation and induces apoptosis of chronic myeloid leukemia cells via targeting IL6/JAK/STAT3 pathway. Exp Ther Med.

[48] Sun K, Luo J, Guo J, Yao X, Jing X, Guo F (2020). The PI3K/AKT/mTOR signaling pathway in osteoarthritis: a narrative review. Osteoarthr Cartil.

[49] Alzahrani AS (2019). PI3K/Akt/mTOR inhibitors in cancer: at the bench and bedside. Semin Cancer Biol.

[50] Mallak AJ, Abbaszadegan MR, Khorasanizadeh PN, Forghanifard MM (2016). Contribution of EVX1 in aggressiveness of esophageal squamous cell carcinoma. Pathol Oncol Res.

[51] Xiao X, Dai Z, Liu F, Zhao X, Wu Q (2025). KLF9 in cancer: a potential prognostic marker and therapeutic target. Front Oncol.

[52] Yuce K, Ozkan AI (2024). The kruppel-like factor (KLF) family, diseases, and physiological events. Gene.

