# Supplementary material for: A multi-dataset associative analysis of single-cell and bulk transcriptomic profiles in preeclampsia
Source: Open Med (Wars). 2026 Oct 26;21(1):20261529. doi: 10.1515/med-2026-1529 (PMC13695452; doi:10.1515/med-2026-1529)
Supplement: Supplementary file 1 [file j_med-2026-1529_suppl_001.docx]

fx1

Supplementary Figure S1: Preprocessing of Single-Cell Data (A) Single-cell quality control displays the number of cells, genes, and sequencing depth for each sample.en

(B) The left plot illustrates the relationship between cell sequencing depth and mitochondrial content. The middle plot depicts the correlation between mitochondrial content and nCount_RNA, while the right plot shows the connection between sequencing depth and the number of genes. In the scatter plot, the y-axis represents mitochondrial content, and the x-axis represents nCount_RNA. Each point corresponds to a cell, highlighting the distribution of RNA counts in relation to mitochondrial gene expression levels.en

(C) We identified genes exhibiting significant variation across cells and created a feature variance plot.en

(D) A ranked plot of variance for each principal component (PC).en

fx2(E-F) The visualization of PCA (Principal Component Analysis) displays the distribution of principal components (PCs), with each point representing a cell and colors distinguishing different samples.en

Supplementary Figure S2. Assessment of sample-associated variation before and after Harmony correction. (A) UMAP visualization generated from the uncorrected PCA dimensions and colored by individual sample.en

(B) UMAP visualization generated from the uncorrected PCA dimensions and colored by study group.en

(C) UMAP visualization generated from the Harmony-corrected embeddings and colored by individual sample.en

(D) UMAP visualization generated from the Harmony-corrected embeddings and colored by study group. Harmony correction improved the mixing of cells from different samples within several major transcriptional populations. However, because the dataset included only four individuals and sample identity was closely associated with disease status, residual technical, inter-individual, and disease-associated variation could not be completely separated.en

fx6

Supplementary Figure S3. Expression and within-dataset ROC analyses of EVX1 and KLF9 in GSE74341. (A) Comparison of EVX1 and KLF9 expression between preeclampsia and control samples in GSE74341. Group differences were assessed using the Wilcoxon rank-sum test.en

(B) Receiver operating characteristic curves for EVX1 and KLF9 within GSE74341. The AUC values were 0.807 for EVX1 and 0.800 for KLF9. These analyses were performed within the same dataset and should not be interpreted as independent validation.en

fx12

Supplementary Figure S4: Immune- and metabolism-related pathway scores associated with EVX1 and KLF9.en

Dot size indicates statistical significance, and color indicates the direction and magnitude of the estimated difference in pathway scores.en
