# Supplementary material for: A multi-dataset associative analysis of single-cell and bulk transcriptomic profiles in preeclampsia
Source: Open Med (Wars). 2026 Oct 26;21(1):20261529. doi: 10.1515/med-2026-1529 (PMC13695452; doi:10.1515/med-2026-1529)
Supplement: Supplementary file 2 [file j_med-2026-1529_suppl_002.docx]

| **Cluster(s)** | **Final annotation** | **Representative marker genes used for annotation** | **Annotation evidence** | **Potential ambiguity** |
| --- | --- | --- | --- | --- |
| 0 | Granulocyte | S100A8, FCGR3B, SELL | Strong expression of canonical granulocyte-associated markers | No major mixed identity observed |
| 1, 6, 11 | Macrophages/Monocytes | CD14, CD163, CD68 | Expression of monocyte/macrophage lineage markers; supported by SingleR, CellMarker and PanglaoDB | Macrophage and monocyte states were combined at the major-cell-type annotation level |
| 2, 4 | Villous cytotrophoblast cell | MET, CDH2, CDH1 | Expression of trophoblast/epithelial-associated markers | Multiple transcriptional states were collapsed into one major villous cytotrophoblast category |
| 3 | Myelocyte | TCN1, CEACAM8, S100A9 | Expression of immature myeloid/granulocytic markers | Partial overlap with granulocyte-associated genes may occur |
| 5 | Extravillous trophoblast cell | HLA-G, PAPPA2, MMP11 | Strong expression of canonical extravillous trophoblast markers | No major mixed identity observed |
| 7, 12, 13 | B cell | CD79A, CD79B, CD19 | Expression of canonical B-cell markers | Small cluster sizes may reduce annotation robustness |
| 8, 9 | T/NK cell | CD3D, CD3G, GZMA | Expression of T-cell and cytotoxic lymphocyte markers | T and NK cells were combined because of overlapping transcriptional features |
| 10 | Syncytiotrophoblast cell | CYP19A1, CSH1, ERVFRD-1 | Expression of canonical syncytiotrophoblast-associated genes | No major mixed identity observed |

Supplementary Table S1. Cluster-level annotation and representative marker genes
